# Surgical Treatment of Atrial Fibrillation: An Update

**DOI:** 10.21470/1678-9741-2025-0903

**Published:** 2025-08-22

**Authors:** Vinicius José da Silva Nina, Henrique Murad, João Ferreira Leal, Fernando Antonio Lucchesi, Rui M. S. Almeida, Gustavo Ieno Judas, Paulo Roberto Slud Brofman, Carlos Manuel de Almeida Brandão, Luiz Cláudio Moreira Lima, Valquíria Pelisser Campagnucci

**Affiliations:** 1 Universidade Federal do Maranhão, Hospital Universitário Presidente Dutra, São Luís, Maranhão, Brazil; 2 Universidade Federal do Rio de Janeiro, Hospital Universitário Clementino Fraga Filho, Rio de Janeiro, Rio de Janeiro, Brazil; 3 Department of Surgery, Faculdade de Medicina de São José do Rio Preto (FAMERP), São José do Rio Preto, São Paulo, Brazil; 4 Cardiovascular Surgery Division, Hospital São Francisco, Santa Casa de Misericórdia de Porto Alegre, Porto Alegre, Rio Grande do Sul, Brazil; 5 Instituto de Cirurgia Cardiovascular, Faculdade de Medicina, Centro Universitário Fundação Assis Gurgacz, Cascavel, Paraná, Brazil; 6 Sociedade Brasileira de Cirurgia Cardiovascular (SBCCV), São Paulo, São Paulo, Brazil; 7 Centro de Cardiomioplastia Celular, Pontifícia Universidade Católica do Paraná (PUCPR), Curitiba, Paraná, Brazil; 8 Cardiovascular Surgery Division, Instituto do Coração do Hospital das Clínicas da Faculdade de Medicina da Universidade de São Paulo (InCor-HCFMUSP), São Paulo, São Paulo, Brazil; 9 Hospital Madre Teresa, Belo Horizonte, Minas Gerais, Brazil; 10 Faculdade de Ciências Médicas da Santa Casa de São Paulo, São Paulo, São Paulo, Brazil

**Keywords:** Atrial Fibrillation, Atrial Appendage, Surgical Instruments, Vascular Diseases.

## Abstract

In this article, the authors present the indication for surgical ablation of atrial fibrillation and of left atrial appendage occlusion. They also present technical aspects of Cox-Maze IV operation and of left atrial appendage clip occlusion. They discuss the result of those techniques and what the guidelines recommend for their use.

## INTRODUCTION

**Table:** 

Abbreviations, Acronyms & Symbols				
AADs	= Antiarrhythmic drugs		LA	= Left atrium
AF	= Atrial fibrillation		LAA	= Left atrial appendage
BFR	= Bipolar radiofrequency		LOE	= Level of evidence
CABG	= Coronary artery bypass grafting		PVI	= Pulmonary vein isolation
CM-III	= Cox-Maze III		PVs	= Pulmonary veins
CM-IV	= Cox-Maze IV		RA	= Right atrium
CPB	= Cardiopulmonary bypass		RAA	= Right atrial appendage
CS	= Coronary sinus		SA	= Surgical ablation
HRS	= Heart Rhythm Society		STS	= Society of Thoracic Surgeons
IS	= Intercostal space		SVC	= Superior vena cava
IVC	= Inferior vena cava		TA	= Tricuspid annulus

Atrial fibrillation (AF) is defined as a supraventricular tachyarrhythmia with uncoordinated atrial activation and, consequently, ineffective atrial contraction^[^1^]^. The irregular and disorganized rhythm in the atria of the heart, characteristic of AF, is associated with a significant increase in the risk of stroke, dementia, heart failure, and death^[^2^]^.

AF is one of the most prevalent cardiac arrhythmias, affecting 33,5 million people worldwide and more than 1,2 million Brazilians. Approximately 170,000 new cases of AF are recorded each year in Brazil^[^3^,^4^]^. From 2008 to 2021, there were 406,666 hospitalizations in Brazil due to AF, generating total costs, after adjustment for Brazilian inflation, of R$569,678,472.00^[^3^,^5^]^.

Data from the Estudo Longitudinal de Saúde do Adulto ELSA-Brasil population-based study show a prevalence of AF of 0.3% (men, 0.5%; women, 0.2%). This prevalence is highly associated with advancing age, reaching 7.0% in octogenarians (8.4% in men *vs.* 5.9% in women), and with other risk factors, such as hypertension, heart failure, coronary artery disease, valvular heart disease, obesity, diabetes mellitus, obstructive sleep apnea, and chronic kidney disease^[^5^,^6^]^. Another risk group is patients presenting for cardiac surgery, 11% of whom have a known history of AF. This incidence rises to 30% to 50% in patients who are candidates for mitral valve surgery^[^1^]^.

The goals of AF treatment include ventricular rate control, rhythm control, and prevention of thromboembolism. Therapeutic modalities include the use of antiarrhythmic drugs (AADs), electrical cardioversion, catheter ablation, and surgical ablation (SA)^[^2^]^. This update aims to review the state of the art of SA with emphasis on the Cox-Maze IV (CM-IV) procedure associated with the exclusion of the left atrial appendage (LAA) in those patients refractory to other therapeutic modalities^[^1^,^5^]^.

## HISTORICAL EVOLUTION OF SURGICAL ABLATION FOR ATRIAL FIBRILLATION

The first surgical approaches to the treatment of AF consisted of left atrial isolation, also known as the corridor procedure, and atrial transection. Both techniques were able to control the irregular rapid rate, but by leaving one or both atria in fibrillation, they could not correct the problems of loss of atrial contraction and risk of thromboembolism.

In 1986, Cox and collaborators improved the atrial transection that later culminated in the maze procedure^[^7^]^. This procedure is designed to interrupt any and all macroreentrant circuits, while at the same time directing the sinus impulse along a specific route, thus preserving the functions of the sinoatrial node and atrial conduction. Subsequent modifications were developed to address the problems of transient sinus node dysfunction and left atrial dysfunction resulting in what became known as the Cox-Maze III (CM-III) procedure, also called “cut-and-sew,” becoming the gold standard compared to previous techniques.

Although CM-III is effective for permanent ablation of AF, it has not been widely adopted due to operative complexity, invasiveness, and morbidity (bleeding, cardiac rupture, coronary occlusion)^[^8^]^. The emergence of devices and energy sources such as radiofrequency and cryotherapy allowed the development of a less complex operation, giving rise to the CM-IV procedure, which quickly became the most common SA modality for AF today, being used in > 60% of patients with AF who require concomitant mitral valve surgery. These thermal energy source devices function as alternatives to surgical incisions, and their success depends on the ability to recreate the continuity, linearity, and transmurality of the surgical lesions performed in CM-III^[^9^]^.

## INDICATIONS FOR SURGICAL ABLATION FOR ATRIAL FIBRILLATION

SA of AF may be indicated primarily alone or concomitantly with other cardiac surgeries.

### Isolated Primary Surgical Ablation

According to the 2023 Society of Thoracic Surgeons (STS) Clinical Practice Guidelines for Surgical Treatment of Atrial Fibrillation, isolated primary SA is a reasonable procedure for symptomatic AF in the absence of structural heart disease refractory to class I/III AADs and/or catheter ablative therapy (class IIA, level of evidence [LOE] B-R)^[^10^]^. The 2017 Heart Rhythm Society (HRS) Consensus also considers the same class IIA indication for isolated primary SA in those patients who prefer a surgical approach rather than catheter ablation, provided that refractoriness or intolerance to at least one class I or class III AADs is demonstrated^[^11^]^.

Both societies (STS and HRS) recommend CM-III or CM-IV instead of pulmonary vein isolation (PVI) for persistent or long-standing persistent AF (class IIA, LOE B-NR) and do not recommend PVI alone in the presence of more than moderate mitral regurgitation (class III, LOE-C). The STS guidelines also do not recommend PVI alone if the left atrial dimension is ≥ 4.5 cm (class III, LOE-C)^[^10^,^11^]^.

### Surgical Ablation Concomitant with Other Operations

The STS 2023 guidelines recommend SA for AF at the time of concomitant mitral valve operations (class I, LOE A) or for any other primary elective cardiac surgery (first surgery) with the aim of restoring sinus rhythm and improving long-term outcomes (class I, LOE B-NR)^[^10^]^.

### Left Atrial Appendage Occlusion

The LAA is a potential source of cerebral emboli in patients with AF. For these patients, long-term reduction in stroke risk comes from restoration of sinus rhythm, elimination of the LAA, and/or restoration of atrial contraction. LAA excision/exclusion is recommended both in primary SA alone (HRS consensus LOE B-R) and in SAs concomitant with other elective primary cardiac operations (STS, class I, LOE-A, and HRS consensus LOE C-EO guidelines)^[^10^,^11^]^.

## OPERATIVE TECHNIQUE: COX-MAZE IV

The Cox-Maze procedure is considered the most effective surgical treatment for AF. Its main goals include:

a) Restoring normal sinus rhythm.
b) Preventing stroke by excluding the LAA.

CM-IV uses energy sources to create ablation lines that replicate the CM-III cut-and-sew lesions using bipolar radiofrequency (BRF) and cryotherapy^[^12^,^13^]^. The approaches to the CM-IV are the median sternotomy for AF ablation concomitant with another cardiac operation and the right minithoracotomy approach for those cases in which primary SA alone is sufficient.

### Cox-Maze IV by Sternotomy

Concomitant SA can be classified as “open”, in which a left atriotomy is an integral part of the primary operation, such as mitral valve repair or replacement (with or without tricuspid valve repair), or “closed”, in which a left atriotomy is not necessary for the primary procedure, *e.g.*, coronary artery bypass grafting (CABG) +/- aortic valve replacement.

“Open” SA allows for the full set of CM-IV lesions, which consists of left atrial and right atrial lesions as follows:

### Left Atrial Lesion (L)

#### L1-4: Isolation of the posterior left atrium - “box lesion”

**Fig. 1:**
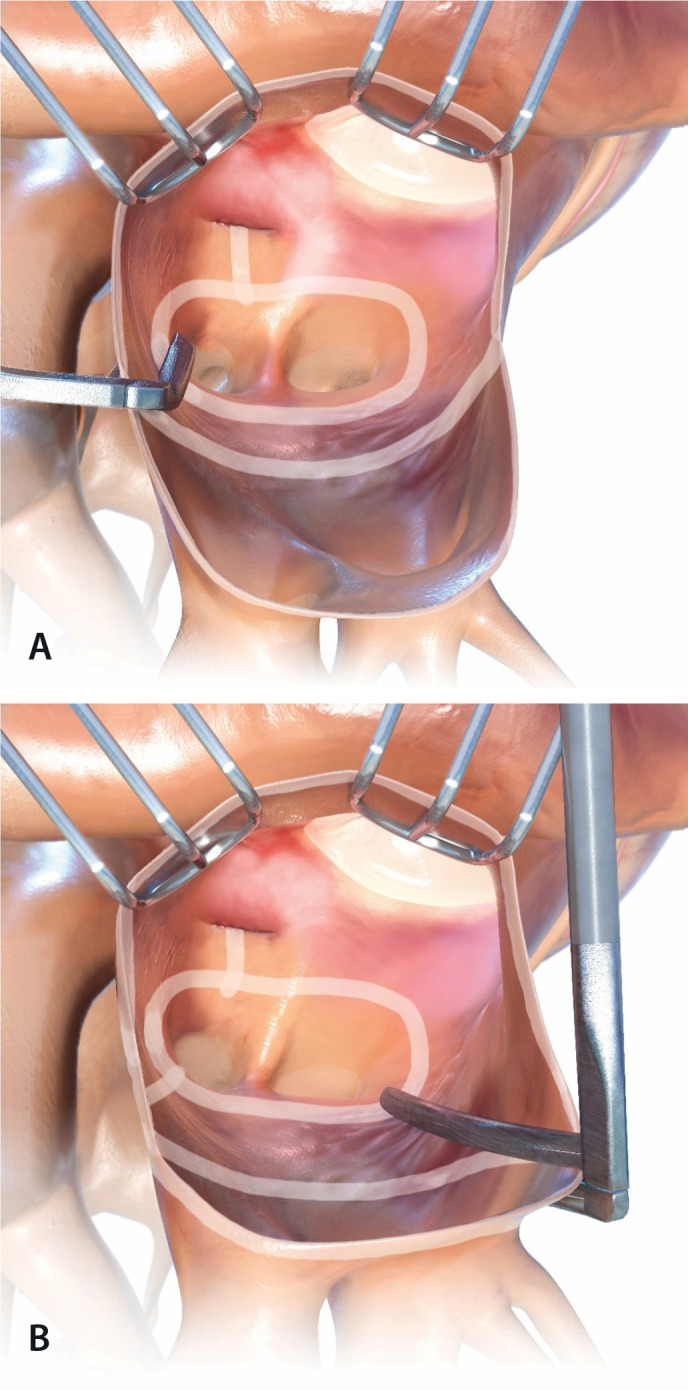
Left atrial isolation (“box lesion”).

The box lesion consists of performing four lesions to electrically isolate the entire posterior part of the left atrium (LA), covering all the pulmonary veins (PVs) and the posterior left atrial tissue between the PVs. In this way, two separate ablation lesions are created - one around the left PVs and one around the right PVs. These vertical lesions are connected to each other by means of two horizontal lines forming a “box”: left atrial roof line (connecting superior PVs) and left atrial floor line (connecting inferior PVs) (Figure 1).

#### L5: Mitral isthmus line (including coronary sinus)

**Fig. 2:**
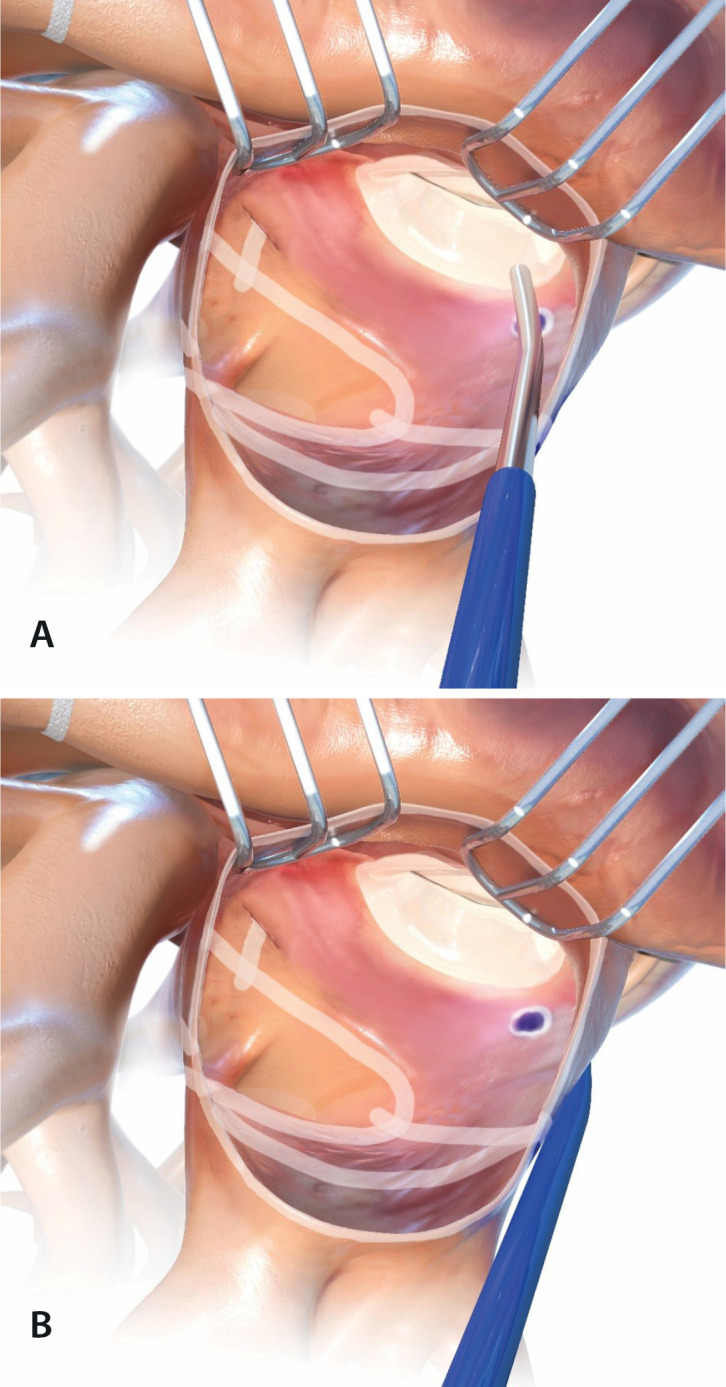
Mitral isthmus line (a) including coronary sinus (b).

This step consists of creating a linear lesion connecting the left atrial isolation box to the mitral annulus, which is electrically nonconductive. This lesion is essential to prevent typical and atypical atrial flutter. The coronary sinus (CS) should be ablated at this time to prevent aberrant conduction of electrical signals that lead to atrial flutter around the mitral annulus region (Figure 2).

Left atrial isolation is completed by the occlusion of the LAA. LAA occlusion techniques include excision and suture, internal ligation, and external exclusion applied by devices such as staplers, endoloops, and by specially designed devices such as the Food and Drug Administration-cleared AtriClip® LAA Exclusion System (AtriCure Inc.) that, in addition to exclusion, electrically isolates the LAA^[^14^]^. Excision and application of an epicardial exclusion device such as the AtriClip® have been shown to be the most effective means of eliminating the LAA. However, in both techniques, it is important not to leave a stump, because residual LAA tissue has been shown to be prothrombotic^[^13^]^.

**Fig. 3:**
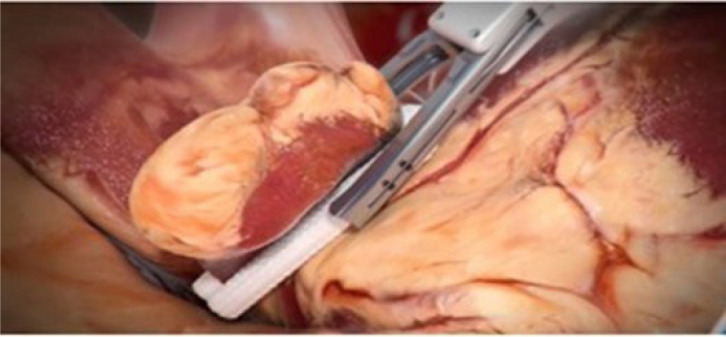
Epicardial occlusion of the left atrial appendage.

When the epicardial clip is chosen, care must be taken under direct vision when applying it to the base of the LAA, keeping the stump < 10 mm, but avoiding the circumflex coronary artery. The application of AtriClip® should preferably be done with the LA open because the LAA will be decompressed, facilitating its application^[^15^]^ (Figure 3).

### Right Atrial Lesion (R)

Right atrial ablation is performed by creating six lesions with the aim of creating a complete line of blockade from the superior vena cava (SVC) to the inferior vena cava (IVC) connecting this line to the native non-conducting tissue of the tricuspid annulus (TA) and this, in turn, to the right atrial appendage (RAA).

The ablation lines are as follows:

#### R1: Right atriotomy

**Fig. 4:**
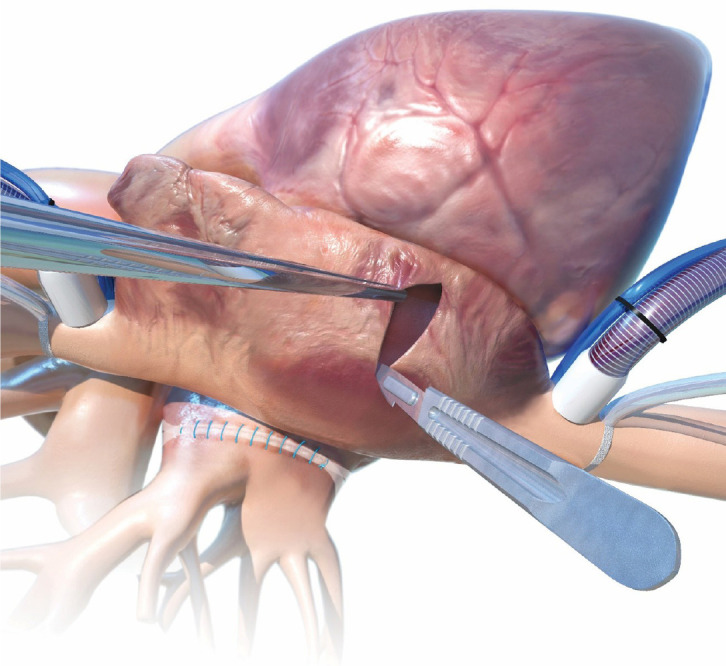
Right atriotomy.

The right atriotomy should be oriented relatively perpendicular to the axis of the SVC and IVC. The atriotomy, being transmural, is electrically nonconductive (Figure 4).

#### R2-3: Lesions of vena cava

**Fig. 5:**
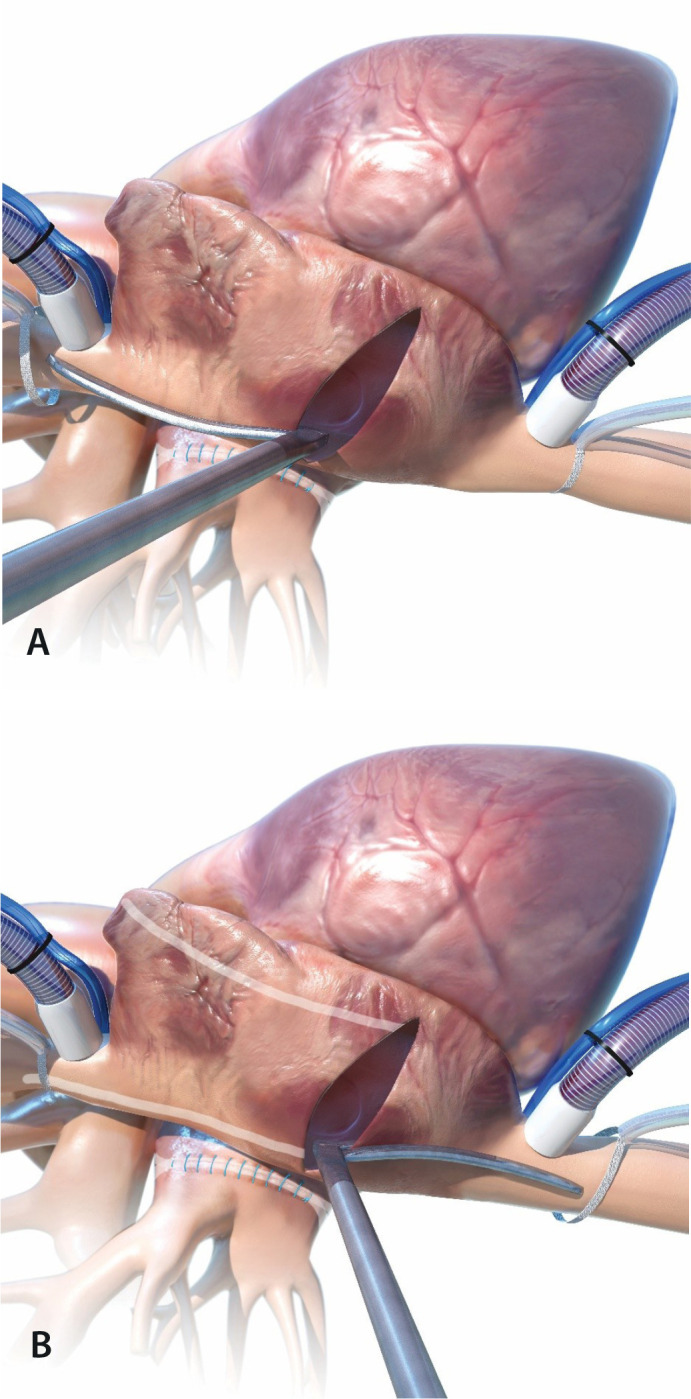
Lesions of vena cava.

Vena caval lesions form a continuous straight line that is perpendicular to the atriotomy connecting the SVC (R2) and the IVC (R3). These lesions are placed as far posterior and lateral as possible to avoid injury to the sinus node. These lesions should extend several centimeters into the vena cava to ensure that the line begins and ends entirely in electrically nonconductive tissue (Figure 5).

#### R4: Cavotricuspid isthmus line

**Fig. 6:**
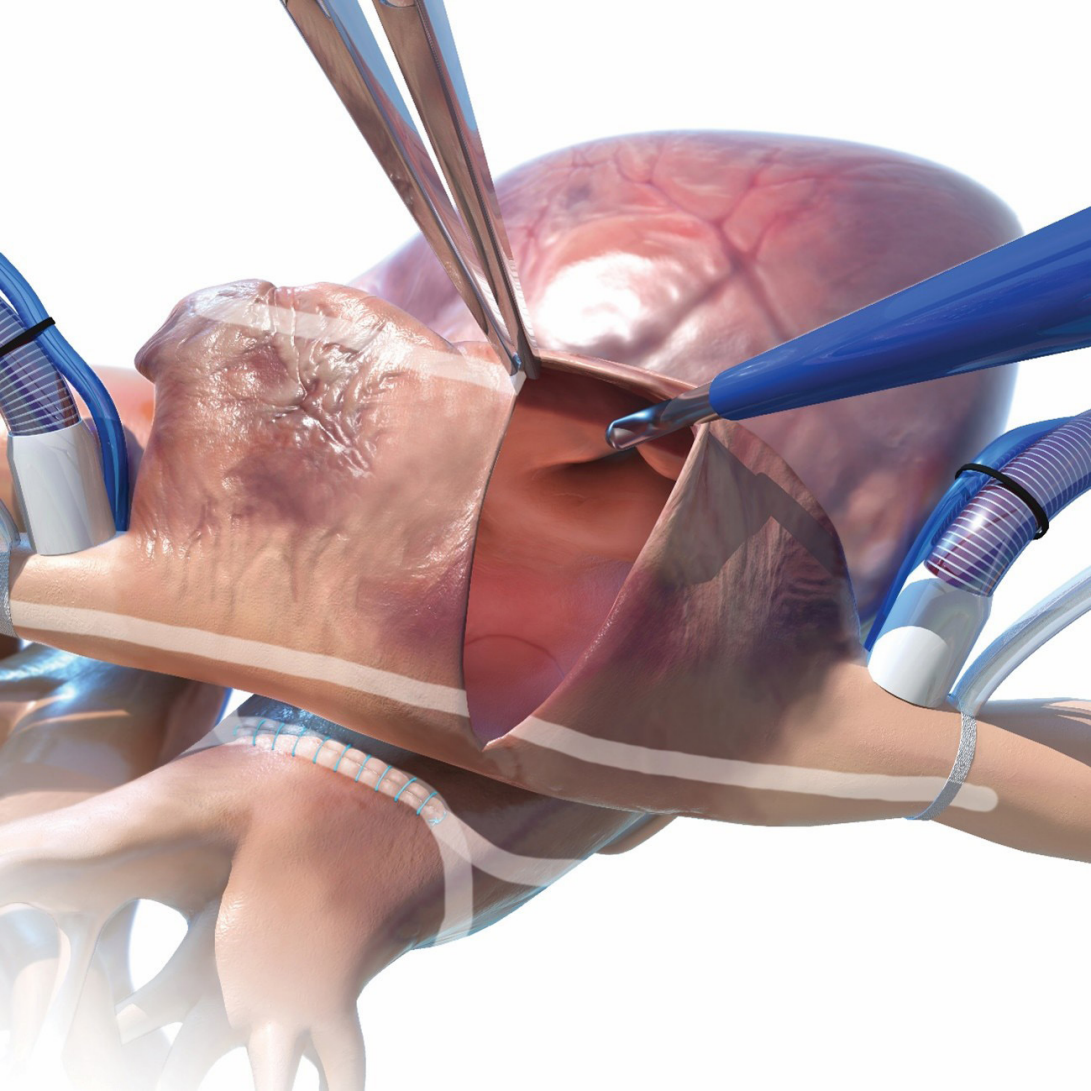
Cavotricuspid isthmus line.

Right atriotomy (free wall) connecting line to the TA at the two o'clock position relative to the valve. This connecting line anchors the anterior right atrial lesions (R1-3) to the electrically inactive tissue of the TA (Figure 6).

#### R5: Connecting line from the tricuspid annulus (10 o'clock position) to the right atrial appendage

**Fig. 7:**
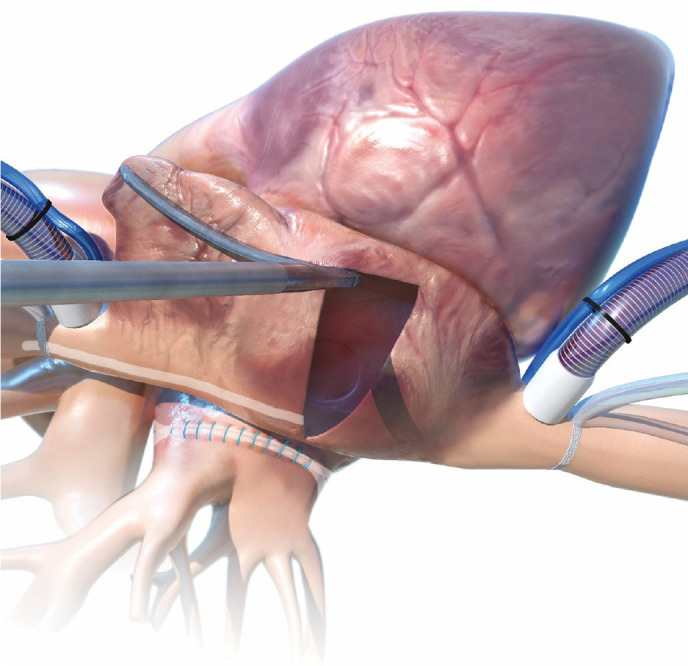
Connecting line from the tricuspid annulus (10 o'clock position) to the right atrial appendage.

This lesion completes the line of block through the right atrium (RA), connecting the TA to the RAA. This lesion prevents rotation of macroreentrant circuits around the RAA and may be particularly important in patients with enlarged RA (Figure 7).

#### R6: Connecting line of the right atrial appendage to the free wall of the right atrium

Additional lesion of the base of the RAA to the free wall of the RAA on the aortic side of the RAA to avoid the sinoatrial node.

### Cox-Maze IV by Right Minithoracotomy

In isolated AF, there is an increasing trend toward minimally invasive SA. The complete set of CM-IV lesions can be performed through a right minithoracotomy with cannulation of the femoral vessels for cardiopulmonary bypass (CPB). In evaluating patients for minithoracotomy, preoperative transesophageal echocardiography is mandatory to rule out contraindications, including left atrial or LAA thrombus, giant LA, or mitral regurgitation. Other contraindications include lung disease that precludes one-lung ventilation, coronary artery disease, prior cardiac surgery, and dense pleural adhesions. In the presence of these contraindications, open SA is mandatory^[^1^,^9^,^16^]^.

#### Access Route

**Fig. 8:**
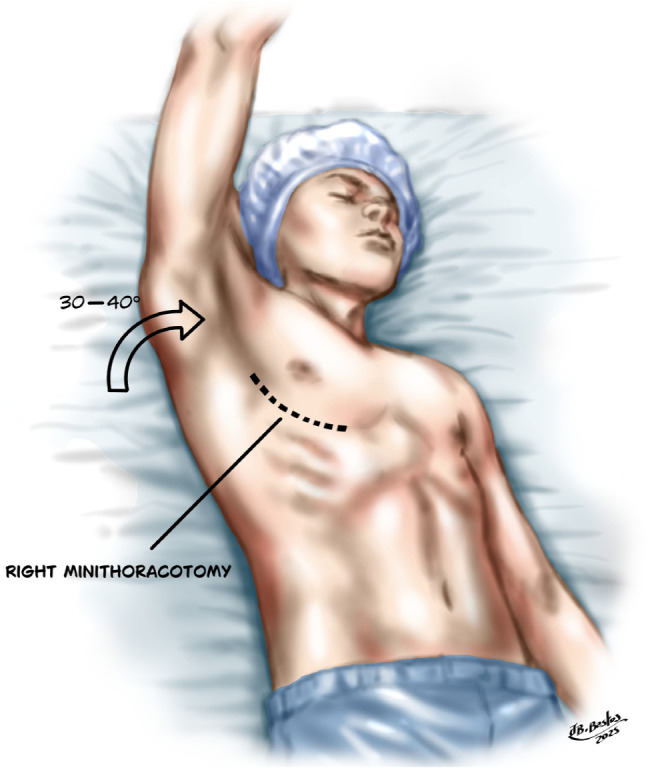
Preparation for Cox-Maze IV procedure via right minithoracotomy and cannulation of the femoral vessels.

A 4 - 5 cm anterolateral minithoracotomy is performed at the third intercostal space (IS). The camera port is positioned at the second IS in the anterior axillary line. The right pericardium is opened at least 2 cm above the phrenic pedicle to avoid nerve injury. Heparin is administered and CPB is started. The aorta is cross-clamped and cardioplegic solution is infused antegrade. The LA is opened widely toward the oblique sinus and extended toward the CS to shorten the distance between the bottom of the incision and the mitral annulus^[^9^,^16^]^ (Figure 8).

### Left Atrial Lesion Set

#### Mitral isthmus line (including coronary sinus)

**Fig. 9:**
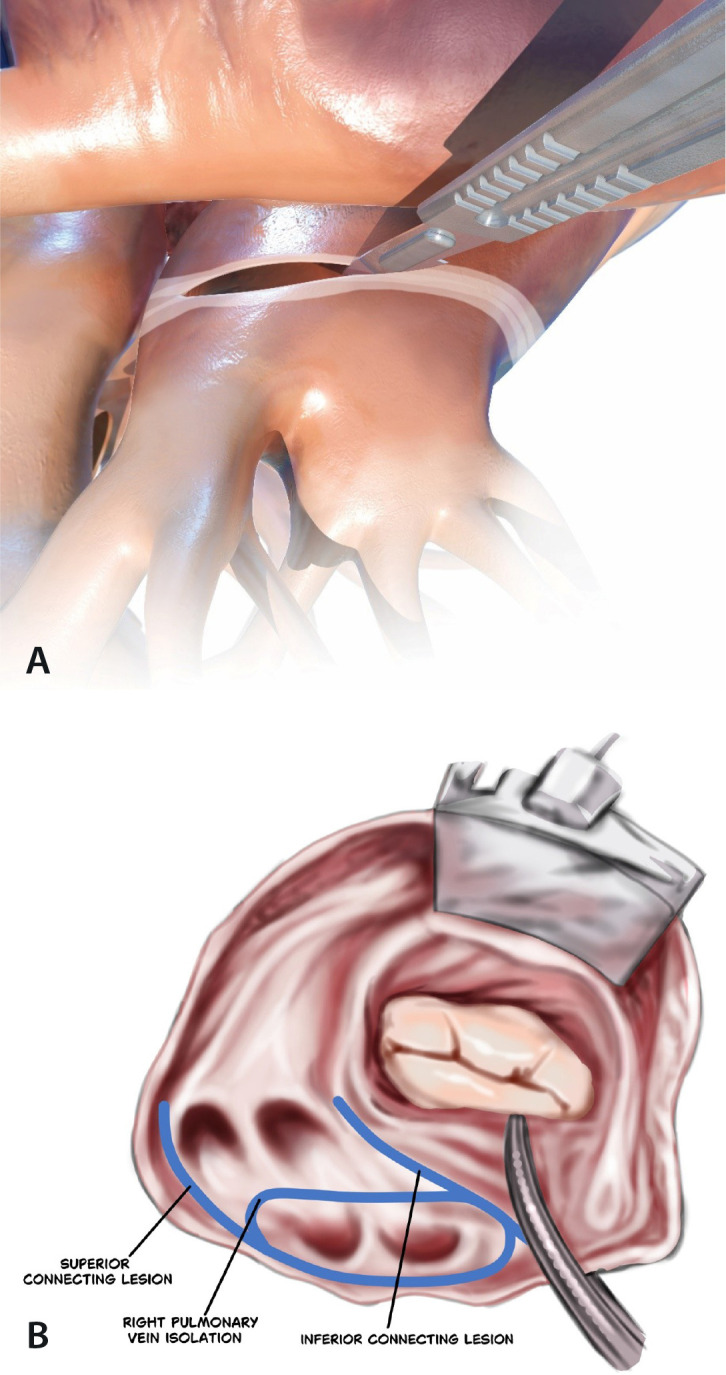
(a) Left atriotomy. (b) Ablation of the mitral isthmus including the coronary sinus.

A BRF articulating forceps is inserted through the left atriotomy with the inner jaw directed toward the mitral annulus, crossing the CS epicardially. This line should be adapted to the coronary anatomy. In left dominance, the ablation should be directed toward the posteromedial commissure of the mitral valve, whereas in right dominance, this line is directed toward the P2 - P3 junction. Alternatively, a cryoprobe is positioned in the epicardium at the level of the CS, overlapping the anterior line of the radiofrequency lesion. A blue marker is used to highlight the endocardial end of the cryotherapy lesion. This helps complete the endocardial ablation line of the mitral annulus posteriorly (Figure 9).

#### Left atrial isolation (“box lesion”)

**Fig. 10:**
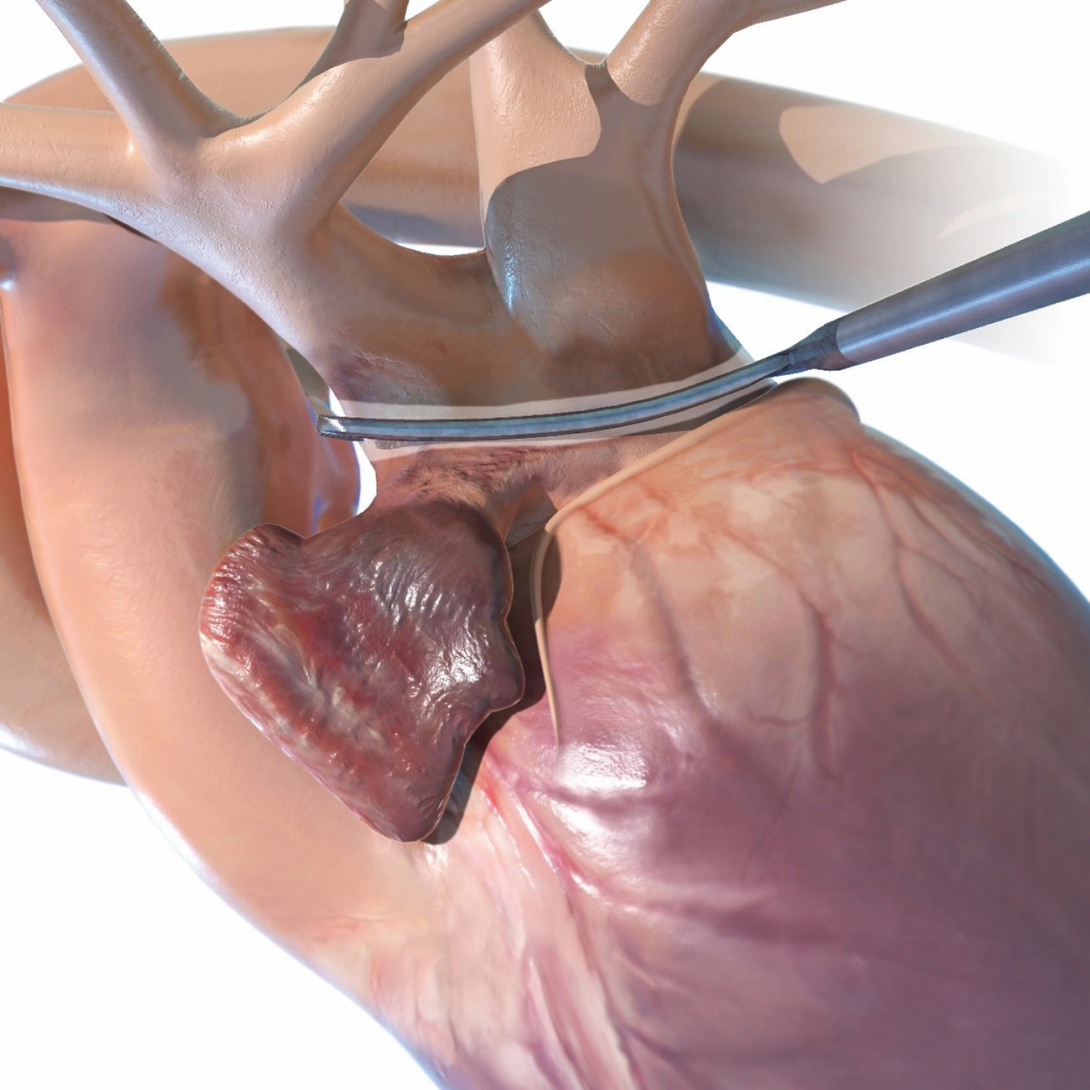
Isolation of the left pulmonary veins for completion of the “box lesion”.

From the inferior border of the left atriotomy, the lower jaw of the BRF forceps is advanced towards the posterior wall of the LA while the upper jaw is introduced into the LAA ostium, thus creating the ablation line of the left atrial “floor”. Similarly, the lower jaw is positioned at the level of the transversus while the upper jaw is advanced from the superior border of the left atriotomy into the LAA ostium, automatically excluding the left PVs and the posterior aspect of the LA, thus creating the ablation line of the left atrial “roof” (Figure 10).

#### Left atrial appendage occlusion

**Fig. 11:**
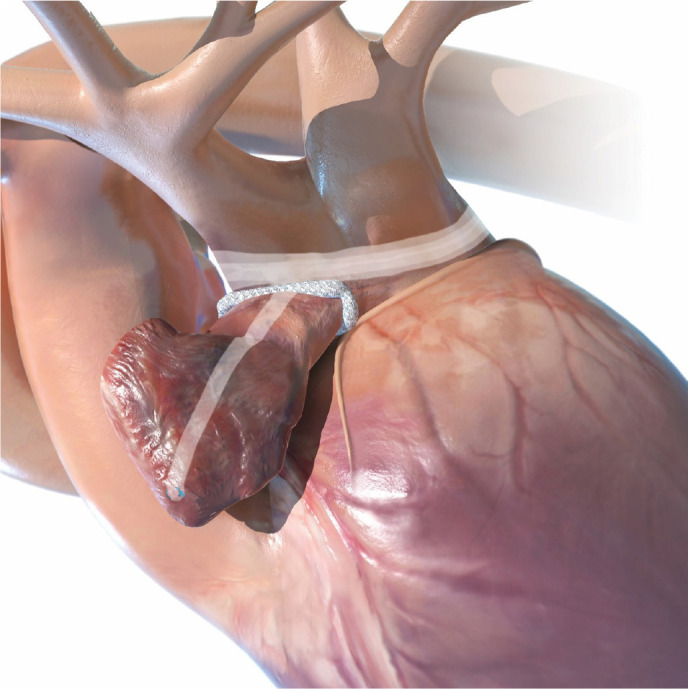
Application of the left atrial appendage exclusion device.

With the RA fully collapsed, the aorta is elevated and the LAA exposed through the transverse sinus. A running 4-0 Prolene® suture at the crest level helps to pull the appendage into the exclusion device (AtriClip® Pro 2). The LAA is then gently mobilized and accommodated until the AtriClip® reaches the base of the LAA (Figure 11).

### Right Atrial Lesion Set

#### Right atriotomy

The RA is opened 1 cm away from and parallel to the right atrioventricular groove. This reduces the distance to the TA.

#### Intercaval line

**Fig. 12:**
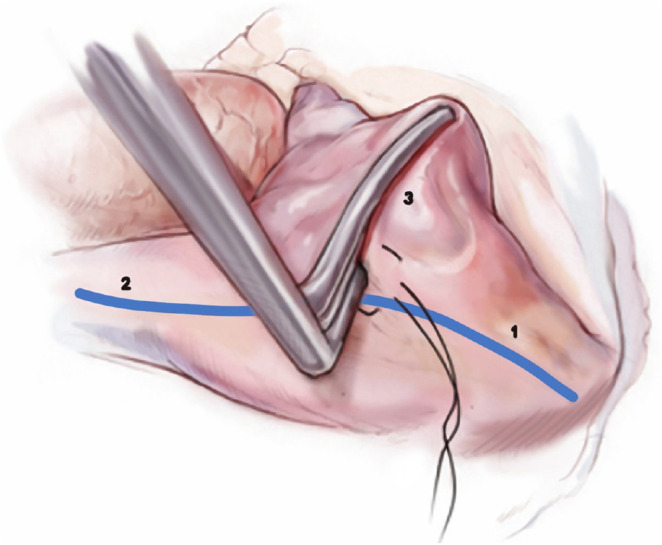
Intercaval and right atrial free wall ablation line (1. inferior vena cava, 2. superior vena cava, 3. right atrial free wall).

A purse-string suture is placed near the Waterston Groove, and through a small incision in the center of the purse, the BRF forceps are introduced toward the SVC with the jaws directed toward the posterior wall to avoid the sinus node. The jaws of the BRF forceps are then inverted and passed along the inferior edge of the right atriotomy toward the IVC, completing the “intercaval line” (Figure 12).

#### Cavotricuspid isthmus line

**Fig. 13:**
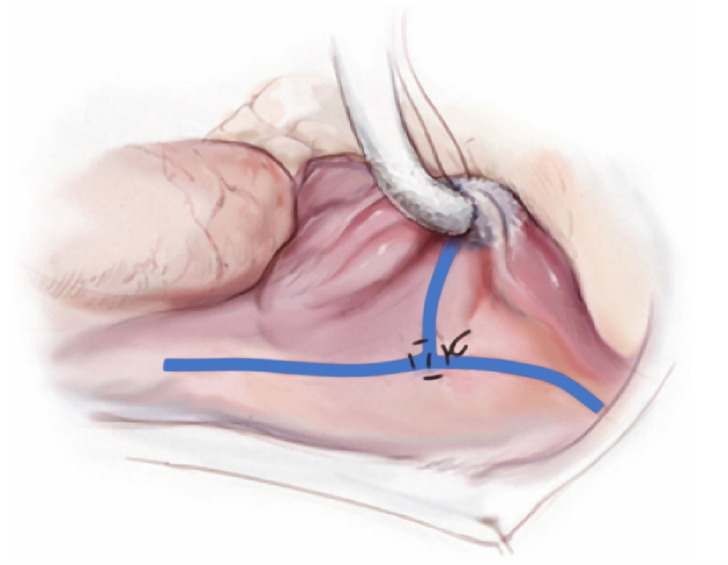
Ablation of the tricuspid isthmus.

The cryoprobe is inserted through the superior border of the right atriotomy and advanced until it reaches the TA at the one to two o'clock position (tricuspid isthmus) (Figure 13).

#### Connection line of the free wall of the right atrium with the atrial appendage

**Fig. 14:**
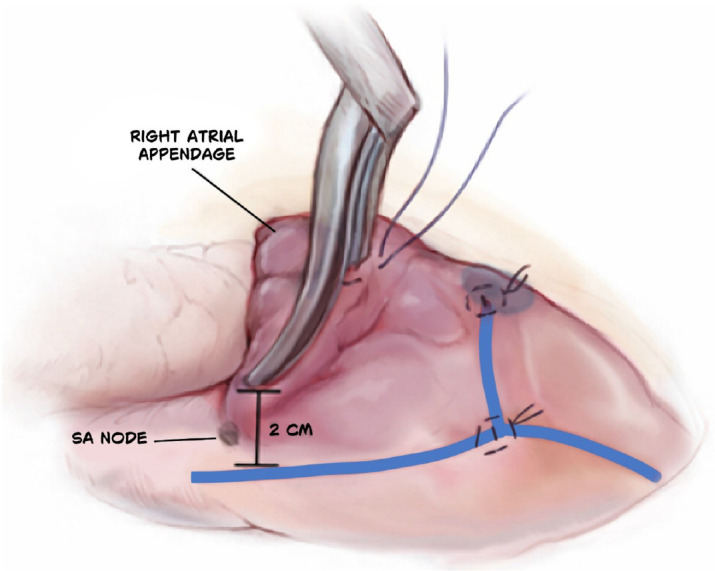
Ablation line connecting the right atrial free wall to the right atrial appendage. SA=surgical ablation.

The BRF clamp is inserted through the upper end of the right atriotomy, advanced toward the tip of the RAA, completing the set of right atrial lesions (Figure 14).

## RESULTS

The results of SA depend on specific criteria that a lesion must meet to consistently and reproducibly block electrical conduction. Even if all CM-IV lesions are made, if any of them do not meet these criteria, the CM-IV as a whole may be incomplete and ineffective. Fortunately, these criteria are simple and are as follows:

1. Each lesion must be transmural throughout its entire length.
2. Each lesion must originate or terminate in tissue that is not electrically conductive^[^13^]^.

In Brazil, SA has been widely studied since the late 1990s and has presented progressively better results as diagnostic advances and ablation technologies are introduced that allow for personalized treatments for patients with AF and expand their applicability^[^17^-^26^]^.

Studies such as those by Ruaengsri et al. demonstrate the safety and efficacy of CM-IV as the gold standard procedure for the treatment of AF with an AF-free success rate of 91% at six months and 93% at 12 months. These authors also demonstrated superiority of CM-IV compared to previous techniques with shorter surgery time, lower complication rate, reduced hospital stay and significant reduction in the risk of postoperative stroke^[^12^]^.

McCarthy and Cox (2024) found similar results with concomitant SA in patients undergoing mitral valve surgery with a 92% AF freedom rate at 12 months and 82% at 36 months and a 33% reduction in stroke risk after LAA occlusion^[^15^]^. These findings allowed the authors to conclude that concomitant SA of AF associated with LAA occlusion reduces the risk of stroke, heart failure, and late mortality, especially in patients undergoing mitral valve surgery.

Malaisrie et al., in turn, evaluated the late results of AF ablation performed during CABG in a cohort of 34,600 Medicare patients linked to the STS database, 10,541 (30.5%) of whom had preoperative AF and received concomitant ablation. In this study, after two years of follow-up, there was a clear benefit for the group that received ablation, with lower mortality (29.9% *vs.* 37.1%, *P* = 0.0358) and incidence of stroke/embolism (9.9% *vs.* 12.0%, *P* = 0.0006)^[^27^]^. Based on these results, the authors concluded that AF ablation concomitant with CABG was able to reduce the risk of mortality and thromboembolic events in long-term survivors (> 2 years) and should be recommended in patients with preoperative AF who present an acceptable perioperative risk.

Regarding the access route, MacGregor et al. found similar efficacy in SA performed by a minimally invasive approach (right mini thoracotomy) compared with median sternotomy^[^28^]^. In both access routes, the most important aspect seems to be the electrical isolation of the LA (“box lesion”). In the study by Robertson et al., the inclusion of the “box lesion” significantly increased the success rate of ablation when compared with isolation of the PVs alone (96% *vs.* 86%)^[^9^]^.

In general, SA has been shown to be a beneficial and safe procedure. The 2023 Clinical Practice Guidelines from the STS highlight the proven benefits of the CM-IV procedure: improved survival and quality of life and a lower incidence of stroke and thromboembolism in the long term. As potential complications, the guidelines highlight that ablation may increase the risks of renal dysfunction and the need for a permanent pacemaker (< 5%), but it does not increase overall mortality^[^10^]^.

## FINAL CONSIDERATIONS

The CM-IV technique is particularly indicated for patients with symptomatic AF who require other concomitant cardiac surgeries, such as valve repair, or for those with AF refractory to medications and catheter ablation. It is also recommended for patients at high risk of stroke, especially in cases where anticoagulation is not feasible. Including LAA management in the procedure is a crucial component, as it significantly reduces the risk of thrombosis and embolic events.

In conclusion, the CM-IV technique represents a milestone in the treatment of AF, combining safety, efficacy, and long-term benefits. Its wider adoption is essential to transform the most severe stages of patients' health, especially in countries such as Brazil, where the prevalence of AF is significant, and the risks associated with this condition severely impact public health. Expanding access to this innovative approach is a crucial step towards improving quality of life and reducing serious complications in patients with AF.
